# Rapid and Sensitive Qualitative Duoplex Real-Time PCR Method for Discriminatory and Confirmatory Diagnosis of HTLV-1 and HTLV-2 Infections: Brazilian Multicentric Study

**DOI:** 10.3389/fmed.2022.881630

**Published:** 2022-06-09

**Authors:** Mauricio Cristiano Rocha-Junior, Evandra Strazza Rodrigues, Svetoslav Nanev Slavov, Tatiane Assone, Maíra Pedreschi, Debora Glenda Lima de La Roque, Maisa Sousa, Viviana Olavarria, Bernardo Galvão-Castro, Benedito Antonio Lopes da Fonseca, Augusto César Penalva de Oliveira, Jerusa Smid, Oswaldo Massaiti Takayanagui, Jorge Casseb, Dimas Tadeu Covas, Simone Kashima

**Affiliations:** ^1^Regional Blood Center of Ribeirão Preto, Ribeirão Preto Medical School, University of São Paulo, São Paulo, Brazil; ^2^Graduate Program in Biosciences and Biotechnology, School of Pharmaceutical Sciences of Ribeirão Preto, University of São Paulo, São Paulo, Brazil; ^3^Center for Cell-Based Therapy CTC, Regional Blood Center of Ribeirão Preto, University of São Paulo, São Paulo, Brazil; ^4^Laboratory of Medical Investigation LIM 56, Division of Dermatology, Medical School, University of São Paulo, São Paulo, Brazil; ^5^Tropical Medicine Center, Federal University of Para, Belém, Brazil; ^6^Centro de HTLV, Escola Bahiana de Medicina e Saúde Pública, Salvador, Brazil; ^7^Department of Neurology, Ribeirão Preto Medical School, University of São Paulo, São Paulo, Brazil

**Keywords:** HTLV-1, HTLV-2, molecular diagnosis, real time PCR, multiplex real time PCR

## Abstract

Human T cell lymphotropic virus (HTLV) is the caustive agent of two main conditions i. e., the HTLV-1-associated myelopathy/tropical spastic paraparesis (HAM/TSP) and the adult T-cell leukemia/lymphoma (ATLL). HTLV diagnosis is based on serological and molecular approaches; however, an accurate and validated method is still needed. The objective of this study was to establish a rapid and sensitive molecular test to confirm and discriminate HTLV 1/2 types. The test validation was performed as a multicentric study involving HTLV confirmation centers throughout Brazil. Proviral DNA was extracted from whole blood and the amplification was performed using in-house designed primer and probe sets targeting the *pol* genomic region. An internal control to validate the extraction and amplification was also included. The limit of detection (LoD) of the assay was four copies/reaction for HTLV-1 and 10.9 copies/reaction for HTLV-2. The diagnostic sensitivity of the platform was 94.6% for HTLV-1, 78.6% for HTLV-2, and the specificity was 100% for both viruses. Cross-reactions of the test with human viruses including HAV, HBV, HCV, HIV-1/2, and parvovirus B19 were not observed. During the multicentric validation, the test was used to screen a total of 692 blood samples obtained from previously confirmed HTLV-positive individuals. From these, 91.1% tested positive being concordant with the previously obtained results. In conclusion, our duoplex-RT-PCR-HTLV1 /2 presented adequate efficiency for HTLV-1/2 differentiation showing high sensitivity and specificity. Therefore, it can be a suitable tool for confirmation of suspected and inconclusive HTLV cases, prenatal and pre-transplant diagnosis, in Brazil and in other countries HTLV-endemic countries.

## Introduction

The human T cell lymphotropic virus type (HTLV) was the first human retrovirus to be identified (1) and four main types are described: HTLV-1 to 4 (2–4), being HTLV-1 and HTLV-2 is the most prevalent. HTLV-1 is associated with an inflammatory neurological disease known as HTLV-1-associated myelopathy (HAM) (5) and a hematological malignancy known as adult T-cell leukemia/lymphoma (ATLL) (6). Furthermore, HTLV-1 has also been associated with several chronic inflammatory syndromes like uveitis, polymyositis, arthritis, alveolitis, infective dermatitis, some types of skin lesions and susceptibility to opportunistic infections (7–9). HTLV-1/2 is transmitted sexually, by sharing of intravenous equipment, by transfusion of cellular blood products, transplantation of organs or tissues, and from a mother to offspring *in utero* prior to labor, during delivery and through breastfeeding (10).

There are ~5–10 million HTLV-infected people worldwide (10). The endemic areas include Southwestern Japan, the Caribbean, Sub-Saharan Africa, South and Central America, Iran, and Melanesia (10). Brazil is considered one of the main endemic areas in South America, where it is estimated that at least 800,000 to 2.5 million people are infected with HTLV-1/2 (11). The HTLV-1 prevalence in Brazil varies according to geographical regions of the country (12), and the majority of cases are present in the urban population while HTLV-2 is more prevalent in indigenous tribes and among intravenous drug users (13, 14).

Since 1993, for transfusion purposes, screening of voluntary blood donors for HTLV-1/2 has been mandatory in Brazil (15). Despite this procedure and the high estimates of HTLV-infected individuals in the country, HTLV infection is still considered a neglected communicable disease since there is no compulsory notification to health authorities. Additionally, the scarcity of accurate diagnostic tests leads to the lack of a validated diagnostic algorithm. Accurate diagnostic tests are a powerful tool to control HTLV transmission.

In Brazil, the recommended algorithm for HTLV-1/2 diagnosis is based on conventional serological methods such as enzyme-linked immunosorbent assay (ELISA), particle agglutination, and chemiluminescence immunoassay. Western blot (WB) and/or line immunoassay (INNO-LIA) are usually applied as confirmatory and discriminatory diagnoses. However, indeterminate WB results can reach approximately 50% (16, 17), leading to an inconclusive diagnosis. In addition, the use of WB and line immune assay is not feasible for many developing countries, mainly due to the high cost. Moreover, ELISA positive or undetermined sera by ELISA were not always confirmed neither by WB nor line immune assays in Brazil.

The use of nucleic acid testing (NAT) platforms for HTLV viral gene detection instead of WB or line immune assay can be applied due to the higher sensitivity and specificity. Several centers have developed their own NAT assays generically called *in-house* tests (18–20). However, there is difficulty in controlling the quality of these tests, particularly because each laboratory uses distinct NAT strategies, such as genome targets, detection methods, and incomplete and/or inadequate methodological validation processes (20–22). These aspects make it challenging to standardize proviral detection limits, as well as to quantify proviral load, which is also an essential parameter for monitoring HTLV-infected individuals. Finally, these studies also showed a low number of samples tested by PCR compared to our study, which restricts the implementation of these molecular tests as routine or confirmatory HTLV diagnosis (18–20). One of the few studies that validated a real time PCR for intrapatient proviral load variability showed that the molecular test is suitable for HTLV diagnosis (23). Additionally there is no confirmatory nucleic acid (NAT) test for HTLV diagnosis which is approved by the Brazilian sanitary.

Thus, we have developed and validated a molecular platform with high sensitivity using a qualitative multiplex real-time PCR (duoplex-RT-PCR-HTLV-1/2) for both screening and discriminatory diagnosis of HTLV-1 and 2 infections. Additionally, this platform has been validated as a multicentric study in four main HTLV centers in Brazil, located in the states of São Paulo (two centers), Bahia, and Pará. The last two are where the majority of HTLV positive cases in Brazil are concentrated.

## Methods

### Samples

A total of 975 samples of whole blood were tested using this current protocol. Among them, HTLV-positive samples belonging to the Neurology Clinic at the School of Medicine of Ribeirão Preto, University of São Paulo (*n* = 145), Institute of Infectious Diseases “Emilio Ribas” (IIER), São Paulo, (*n* = 137), HTLV Reference Center, Bahiana School of Medicine and Public Health, Salvador, Bahia (*n* = 179), and Tropical Medicine Center, Federal University of Pará, Belém, Pará (*n* = 226). Additionally, 198 blood samples were obtained from volunteer blood donors at the Regional Blood Center of Ribeirão Preto at the Faculty of Medicine of Ribeirão Preto and 90 blood samples from HIV, HCV, or HBV infected individuals from the Infectious Diseases Clinic at the Faculty of Medicine of Ribeirão Preto, USP. For the duoplex validation step, we calculated the number of tests that should be performed according to the incidence of HTLV in our region (0.01%) (12). For the multicentric study, we evaluated samples that had been previously tested by each center.

All samples from Ribeirão Preto were previously screened for HTLV-1/2, HIV, HCV or HBV by commercial ELISA such as Murex HTLV I+II (DiaSorin, Darford, United Kingdom), Architect HIV Ag/Ab Combo (Abbott, Wiesbaden, Germany); HCV MONOLISA^®^ (Bio-Rad, CA, United States), and HBV MONOLISA^®^ (Bio-Rad), respectively. The HTLV-1/2 positive samples were confirmed by WB analyses (HTLV Blot version 2.4, Genelabs Diagnostics, Singapore) and by *nested* PCR through amplification of the *tax* and LTR regions for HTLV-1 and pxA, pxB, and LTR regions for HTLV-2 (19). Samples were considered as HTLV positive when they were reactive for ELISA, and WB or *in-house* PCR (12).

### Ethics Statement

The study was approved by the Institutional Ethics Committee at the Clinical Hospital of Ribeirão Preto, School of Medicine of Ribeirão Preto, University of São Paulo (Process No. 12.027/2009) and all participants signed an informed consent form.

### HTLV Positive Controls

The positive control used for HTLV-1 was DNA extracted from the MT-2 cell line (93121518/ECACC), with 2.1 copies of the virus per cell (19). The HTLV-2 positive control was obtained from the Gu cell line (kindly provided by the Rega Institute for Medical Research, Katholieke University, Leuven, Belgium), which harbors 8.3 copies of HTLV-2 per cell (24). Both cell lines were used to the initial steps of duoplex PCR standartization. A single tube of the positive control was produced containing MT-2 and Gu DNA based on spectrophotometer DNA quantification. The range of the positive control standard dilution was from 10^5^ to 10^0^ copies/reaction.

### DNA Extraction

The genomic DNA extraction was performed from peripheral blood by ReliaPrep Blood gDNA Miniprep System DNA isolation kit (Promega, Madison, WI, United States) according to the manufacturer's instructions. The DNA concentration and purity was measured by using Nanodrop 2000c (Thermo Scientific, Delaware, United States).

### Primers and Probe Design

Complete reference genomes of HTLV-1 and−2 (GenBank database accession number: AB513134, AF042071, NC_001436, AF139170, AF033817, AF259264, J02029, D13784, BRRP438, AY563954, AY563953, L03561, U19949, L36905, M86840, AF074966, L02534, GU212854, NC_001488, AF139382, AF074965, AF412314, AF326584, L11456, M10060, AF326583, Y13051, L20734, X89270, Y14365) were aligned using BioEdit v. 7.2.3 software (Tom Hall, Ibis Biosciences, Carlsbad, CA, United States) in order to design primer and probe sets (Table 1) at conserved regions for both HTLV-1 and HTLV-2. Two pairs of primers and two distinct hydrolysis TaqMan^®^ probes were designed for the *pol* gene for HTLV-1 and HTLV-2. The sets of primers and probes were designed to ensure the specific detection of the most important HTLV-1/2 subtypes. Beta globin gene was used as internal amplification control (IAC) which allows for the verification of the extraction efficiency. For the design of the oligonucleotides Primer Express^®^ 3.0 (Thermofisher Scientific, Waltham, MA, United States) and Oligo Analyzer v. 3.1 (Integrated DNA Technologies, Coralville, IA, United States) software were used. Primers and probe sequences are indicated in Table 1.

**Table 1:** *Primers* and probes sequences for HTLV-1, HTLV-2, and beta-globin (internal amplification control) detection.

**ID**	**Sequence (5^**′**^-3^**′**^)**	**Tm (**°**C)**	**GC (%)**	**Length (bp)**
HTLV-1 For_*pol*	CAGCCCCTTCACAGTCTCTACTG	59	57	23
HTLV-1 Rev_*pol*	AGAAGGATTTAAATATATTTGGTCTCGG	58.5	32	28
HTLV-1 probe	*FAM* CCTTACAAAGGCATACTGAT *MGB*	69	40	20
HTLV-2 For_*pol*	CAAGGTGATGTAACCCATTATAAGTACAA	58.8	34	29
HTLV-2 Rev1_*pol*	AACCGCACCGGAGAAGGT	59.1	61	18
HTLV-2 Rev2*_pol*	AGAAACCAGCTGTGAGACTATCAGC	59.1	48	25
HTLV-2 probe	*VIC* AAATACAAATACTGCCTCCACGT *MGB*	68	40	20
Beta-globin For	TGAAGGCTCATGGCAAGAAA	58	45	20
Beta-globin Rev	GGTGAGCCAGGCCATCAC	59	67	18
Beta-globin probe	*NED* TGCTCGGTGCCTTT *MGB*	69.0	63	14

*ID, identification; Tm (°C), melting temperature in Celsius degrees; GC (%), percentage of G and C in the oligonucleotides sequences; bp, base pairs; MGB, minor groove binder. The length of amplicons is 75, 81, and 54 base pairs for HTLV-1, HTLV-2, and beta-globin, respectively*.

### Qualitative Duoplex-RT-PCR-HTLV1/2

#### Optimization of Primer/Probe Concentrations

The assays were optimized initially as *singleplex format*, and then multiplex in order to compare linear correlation (*r*^2^), efficiency of qPCR reactions and shape of the amplification curves. The evaluation of the commercial PCR master mixes applied included intensity of fluorescence (ΔRn), the format of the amplification curve and the appearance of the Ct during the amplification. Distinct concentrations of primers (250–500 nM) and probes (100–300 nM) were used to adjust the best concentration of each. After these initial steps, duoplex-RT-PCR-HTLV1/2 reactions were standardized in a final volume of 25 μl with the reaction mixture consisting of 12.5 μl GoTaq^®^ Probe qPCR Master Mix (Promega) and 5 μl of DNA (0.1 μl/ml, 500 ng), 500 nM for each forward and reverse primer for HTLV-1, and 200 nM of the probe. For HTLV-2 detection, two sets of reverse primers used simultaneously were designed to improve the detection. For instance, 500 nM of the forward primer, 250 nM of the reverse 1 primer, and 250 nM of the reverse 2 primer, and 200 nM of probe were used. The amplification of IAC was performed using 250 nM of each primer and 200 nM of probe. The following cycling parameters were applied: initial activation step at 50°C/2 min, denaturation at 95°C/10 min, followed by 45 cycles at 95°C/15 s and 60°C/1 min. Amplification data acquisition was carried out using ABI Prism 7500 Real-time PCR system (Applied Biosystems, Foster City, CA, United States). For all the runs, positive control (DNA from the MT2 and Gu cell line) and IAC were used. After the steps of standardization, a ready-to-use mix composed of primers, probes, and buffers was produced and sent to the participating centers for the multicentric study. Positive controls were produced using DNA from MT2 and Gu cell lines at 10^3^ viral copies/reaction to obtain a reproducible and robust control. For clinical samples, HTLV-1 and HTLV-viral gene amplification limit Ct is <40 to be considered detectable. The homogeneity and stability of these lots of ready-to-use mix were evaluated before their use. Homogeneity was checked by randomly sampling five aliquots of the mix. Stability was evaluated by freezing and defrizzing the mixes (20 times) and checking at intervals of 120 days.

#### Repeatability (Intra-assay) and Reproducibility (Inter-assay)

The obtained variations for the Ct values were all within the postulated limit of within 5% (18, 25, 26). The inter-assay reproducibility was performed using standard dilutions (MT-2 and Gu cell lines) of 10^5^, 10^4^, and 10^3^ proviral copies/reactions. The qPCR reactions were performed in duplicates of each dilution in three separate runs, using a different batch of reagents manipulated by three different operators in three separate days.

### Validation of the Technical Performance of the Assay

#### Analytical Sensitivity

For the analytical sensitivity or limit of detection (LoD, defined as the minimal quantity of target which can be detected), a stock dilution of 10^5^ viral copies was prepared using MT-2 and Gu cell lines as standards. The stock dilution was submitted to serial dilutions until the 62.5, 31.25, 15.625, 7.81, 3.9, 1.95, and 0.975 proviral copies were obtained. Eight replicates of each dilution were pipetted in three independent assays. *Probit* analysis was used to calculate LoD (95% confidence interval) as supplied by the SPSS version 17.0 software (SPSS Inc., Chicago, IL, United States).

#### Analytical Specificity

To evaluate the analytical specificity, *in vitro* and *in silico* tests were performed. For *in vitro* validation, hepatitis A virus, HBV, HCV, HIV-1, HIV-2, and parvovirus B19 panels obtained from the National Institute for Biological Standards and Control (NIBSC) were tested with our platform. In addition to this, we tested 30 samples from HIV-infected patients undergoing HAART treatment, 30 samples from HBV-infected individuals, and 30 samples from HCV-infected individuals. For *in-silico* validation we performed comparisons of our primers and probes with public databases in order to verify possible similarities with other organisms.

#### Diagnostic SensitivityCt

We evaluated three hundred and twenty samples that showed positive results for HTLV-1/2-serology, WB and/or positive nested-PCR. Among them, 278 were for HTLV-1 positive individuals and 42 were for HTLV-2 positive patients. To calculate the diagnostic sensitivity we used the following formula: diagnostic sensitivity = true positive individuals/(false negative individuals + true positive individuals).

#### Diagnostic Specificity

In order to check this parameter, 288 samples from HTLV-1/2 serologically negative subjects were tested with our platform. The diagnostic sensitivity was calculated using the following equation: diagnostic specificity = true negative individuals (false positive individuals + true negative individuals).

### Precision

For the precision tests, the parameters of repeatability (intra-assay) and reproducibility (inter-assay) were evaluated. The repeatability test was performed from three dilutions of the positive controls (MT-2 + Gu) containing 10^5^, 10^4^, and 10^3^ viral copies/reactions with five replicates of each dilution. Three runs were performed on the same day, using the same batch of reagents and prepared by a single operator.

The reproducibility test was performed with three dilutions of the positive controls (MT-2 + Gu) containing 10^5^, 10^4^, and 10^3^ viral copies/reaction with five replicates of each dilution. Three runs using the optimized test were performed on alternate days, using the same batch of reagents and prepared by three different operators.

### Robustness

The robustness was evaluated by the “chess table” method, in which a positive sample is pipetted alongside a negative sample in the qualitative duoplex-RT-PCR-HTLV1/2 plate.

### Statistical Analyses

Precision analysis, reproducibility (intra-assay), repeatability (inter-assay), and comparison of singleplex and multiplex reaction formats using real-time PCR (qPCR) were evaluated using the mean, standard deviation and the coefficient of variation of the cycle threshold (Ct). The *t*-test was used to assess the stability of controls and reagents. One-way ANOVA test was applied to evaluate the homogeneity and stability of the batch of oligonucleotide mix and positive control. The Probit analysis (SPSS) was used to evaluate the analytical sensitivity of the platform.

## Results

### *Singleplex and Duoplex* Reaction Standardization

In order to evaluate *singleplex* format, we generated a standard curve by serial dilutions from 10^5^ to 10^0^ viral copies/reaction of the MT-2 (HTLV-1) and Gu (HTLV-2) positive controls. The efficiency for the probe and primer sets for HTLV-1, HTLV-2, and internal control (IC) were >95% and the linear correlations (*r*^2^) was higher than 0.99.

**Figure 1:**
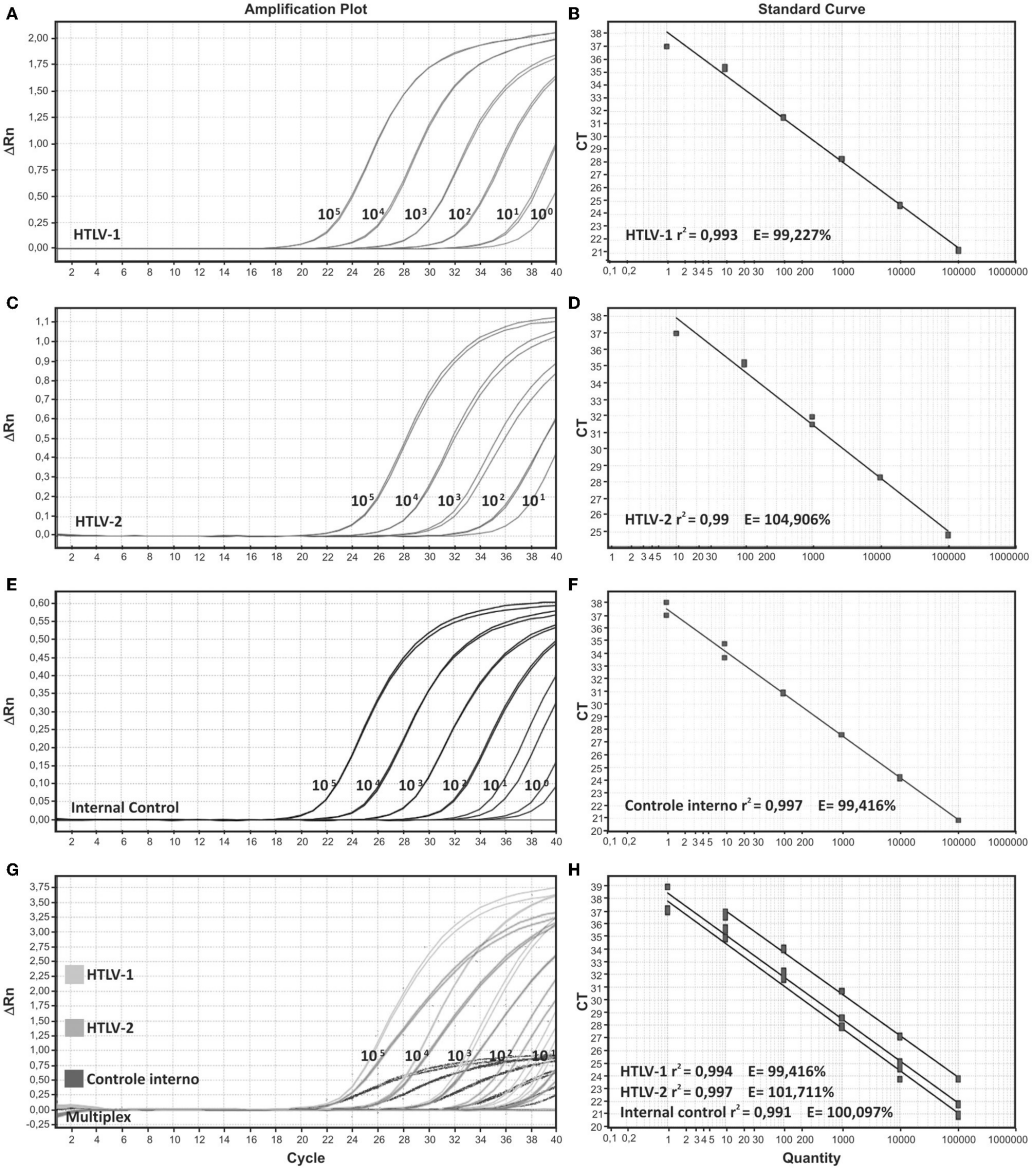
Amplification plots and standard curves of *singleplex* and multiplex assays. *Singleplex* format is demonstrated for **(A,B)** HTLV-1, **(C,D)** HTLV-2, and **(E,F)** internal control, beta-globin. **(G,H)** Multiplex format for the three targets HTLV-1, HTLV-2, and beta globin. Serial decimal dilutions ranging from 105 to 100 copies/reaction were used in the qPCR reactions. DNA from MT-2 and Gu cell lines were used as positive samples for HTLV-1 and HTLV-2, respectively.

In addition, standard curve dilutions were also applied to compare the efficiency and accuracy between the *singleplex* and the *multiplex* qPCR reactions. We observed a high and similar efficiency between the *singleplex* and the *multiplex* format, 93%−100% and 94%, respectively. Correlation (*r*^2^) was also suitable in both formats (0.995–0.996 and 0.997–0.998, for *singleplex* and *multiplex*, respectively) as shown in Figure 1. All tested samples demonstrated positive IAC amplification, thus eliminating PCR inhibition.

### Analytical Sensitivity and Specificity

The 95% LoD of the proposed platform was achieved using two fold serial dilutions (62 to 1 copies/reaction) in eight replicates. The LoD was 4 copies/reaction (range 3–6 copies/reaction) and 11 copies/reaction (range 7–17 copies/reaction) for HTLV-1 and HTLV-2, respectively. Analytical specificity was evaluated *in silico* against public databases (BLAST) to avoid cross-reactivity with other critical human viruses. Primer/probe sequences demonstrated 100% similarity to HTLV-1, HTLV-2, and beta globin (IAC), while *in silico* they did not cross-react with other viral types, making this set of primers/probes suitable for the molecular assay. *In vitro* analytical specificity was evaluated by two strategies: initially, primers/probes were tested on a control panel containing viral genomes obtained from other human pathogens like HAV, HBV, HCV, HIV-1/2, and B19V (National Institute for Biological Standards and Control, NIBSC, London, United Kingdom). This parameter evaluation performed with a total of 90 DNA samples obtained from patients with HIV, HCV, and HBV infections showed 100% specificity, meaning that no cross-reaction with the established diagnostic platform was observed.

### Diagnostic Sensitivity and Specificity of the Assay

From 320 samples tested (278 HTLV-1 and 42 HTLV-2), the duoplex-RT-PCR-HTLV1/2 reached 94.6% of sensitivity for HTLV-1 and 78.6% for HTLV-2. All reactions were performed in duplicate. For calculating the specificity of the assay, 288 samples (198 DNA from blood samples and 90 DNA samples from patients with HIV, HCV, and HBV infections) were tested resulting in no PCR amplification or high Ct values (Ct>40 were considered as negative), therefore demonstrating 100% specificity.

### Repeatability (Intra-assay) and Reproducibility (Inter-assay)

We evaluated the intra-assay using three standard dilutions (10^5^, 10^4^, and 10^3^ copies/reaction) of MT-2 and Gu cells. The coefficient of variation (CV) for Cts values was <5% (Table 2), which is in agreement with the standard validation criteria for the National Virus Reference Laboratory (18, 26). The inter-assay reproducibility was performed using the same standard dilutions (10^5^, 10^4^, and 10^3^ copies/reaction). The multiplex PCR reactions were performed in two replicates of each dilution in three separated runs, using a distinct batch of reagents manipulated by three different operators on three alternate days. The CV for Cts values was within the 5% limit, as illustrated in Table 2. For robustness evaluation (potential for carry-over cross-contamination), we applied the “chess table” method, and HTLV-1 positive samples were pipetted side by side with samples with no nucleic acid targets (NTV). No amplification was detected in any of the NTCs.

**Table 2:** Cycle threshold (Ct) values of repeatability (intra-assay) and reproducibility (inter-assay) analysis of duoplex-RT-PCR-HTLV 1/2 based on standard templates.

**Repeatability (intra-assay)** *						
	**HTLV-1**			**HTLV-2**		
	**10**^**5**^ **copies/reaction**	**10**^**4**^ **copies/reaction**	**10**^**3**^ **copies/reaction**	**10**^**5**^ **copies/reaction**	**10**^**4**^ **copies/reaction**	**10**^**3**^ **copies/reaction**
	***μ*** **±*****σ***	***μ*** **±*****σ***	***μ*** **±*****σ***	***μ*** **±*****σ***	***μ*** **±*****σ***	***μ*** **±*****σ***
Run 1	22.88 ± 0.04	26.31 ± 0.12	29.82 ± 0.10	25.46 ± 0.16	28.92 ± 0.14	32.51 ± 0.22
Run 2	22.57 ± 0.09	25.87 ± 0.07	29.41 ± 0.08	25.76 ± 0.16	29.09 ± 0.15	32.73 ± 0.11
Run 3	22.57 ± 0.08	25.95 ± 0.08	29.68 ± 0.07	25.79 ± 0.16	29.22 ± 0.18	32.86 ± 0.14
μ ±σ	22.67 ± 0.16	26.04 ± 0.22	29.64 ± 0.19	25.67 ± 0.21	29.08 ± 0.20	32.70 ± 0.25
%CV	0.72	0.83	0.65	0.84	0.67	0.76
**Reproducibility (inter-assay)**						
Operator 1	22.44 ± 0.00	25.96 ± 0.01	29.66 ± 0.11	25.79 ± 0.01	28.87 ± 0.06	32.82 ± 0.07
Operator 2	22.45 ± 0.02	25.92 ± 0.02	29.63 ± 0.26	25.73 ± 0.15	28.90 ± 0.06	32.98 ± 0.19
Operator 3	22.49 ± 0.04	25.97 ± 0.03	29.91 ± 0.01	25.89 ± 0.10	29.23 ± 0.05	33.17 ± 0.14
μ ±σ	22.46 ± 0.03	25.95 ± 0.03	29.74 ± 0.19	25.80 ± 0.11	29.00 ± 0.18	32.99 ± 0.19
% CV	0.14	0.11	0.63	0.41	0.63	0.57

* *The repeatability assay was performed in five replicates of each standard dilution in three separated runs, using the same batch of reagents manipulated for a unique operator in the same day*.

### Multicentric Study

After validation steps, we prepared an optimized ready-to-use mix for the duoplex-RT-PCR-HTLV1/2 containing primers, probes, reaction buffer, and positive and internal controls. These pre-aliquoted PCR mixes were distributed to all participating centers of the multicentric study. All centers performed PCR in ABI Prism 7500 real-time PCR System (Applied Biosystem). A total of 692 HTLV samples with previously confirmed diagnosis were tested (Table 3). In Ribeirão Preto, São Paulo, Salvador (Bahia), and Belém (Pará), conventional nested-PCR or real time PCR using tax and LTR gene amplification were the molecular platforms adopted as routine diagnosis. The overall result achieved 91.1% of concordance between duoplex-RT-PCR-HTLV-1/2 and the routine molecular diagnosis adopted in each participating center. The highest concordance was achieved in the Bahia and Para states, 96.5% and 89.3%, respectively for HTLV-1. For HTLV-2, 94.5% and 100% of the concordance was achieved when testing samples from Ribeirao Preto and Bahia. We hightlight that 5.0% of the negative results belonging to suspected cases of HTLV tested positive in duoplex-RT-PCR-HTLV1/2 (Table 3). Negative and positive predictive values were 97% and 89.4%, respectively. All of the participants included in this study signed an informed consent form in their original institutions.

**Table 3:** Multicentric evaluation of the duoplex-RT-PCR-HTLV1/2 platform.

**HTLV Centers**	***n***	**Age (mean)**	**Gender (F|M)**	**HTLV-1**	**HTLV-2**	**Negative**
**(city location)**				**(concordants %)**	**(concordants %)**	**(concordants %)**
Ribeirao Preto^1^	145	38.8	95|47	33/33 (100%)	17/18 (94.5%)	85/94 (91.5%)
Belem^2^	226	43.4	147|79	50/56 (89.3%)	8/13 (61.6%)	149/157 (94.9%)
São Paulo^3^	137	52.6	137|43	78/98 (79.4%)	6/12 (50%)	25/27 (92.6%)
Salvador^4^	179	ND	ND	167/173 (96.5%)	6/6 (100%)	ND

_1_*Regional Blood Center of Ribeirão Preto, Ribeirao Preto Medical School, University of São Paulo, Ribeirao Preto; ^2^Universidade de Belem, Para; ^3^Instituto de Medicina Tropical—University of São Paulo, São Paulo; Medicina Tropical (IMT-USP)—São Paulo; ^4^Escola Bahiana de Medicina e Sayde Publica, Salvador, Bahia*.

*n, number of samples tested; F, female; M, male; ND, non-determined*.

## Discussion

In the current study, we optimized and validated a qualitative *multiplex* qPCR platform—duoplex-RT-PCR-HTLV1/2—for the simultaneous detection and discrimination of HTLV-1 and HTLV-2. The established assay is sensitive and reproducible, easy to perform in molecular biology laboratories with appropriate conditions for clinical diagnosis; it is essentially a low-cost operation compared to the available confirmatory methods such as WB and line immunoassay.

Human T cell lymphotropic virus molecular diagnosis is a significant challenge in regard to standardization, appropriate verification, and validation. Several PCR methods have been reported for HTLV-1/2 diagnosis, but the complete validation procedure was not achieved (18, 25–27). Herein, we followed the main validation steps including analytical and diagnostic sensitivity, analytical and diagnostic specificity, robustness, and precision. Our duoplex-RT-PCR-HTLV1/2 platform demonstrated 94.6% of diagnostic sensitivity for HTLV-1 and 78.6% for HTLV-2 detection. Other studies that establish quantitative PCR demonstrate variable sensitivity. For example, Estes and Sevall (25) demonstrated sensitivity of 81.2% and 87.5% for HTLV-1 and for HTLV-2, respectively. Waters et al. (18) described a sensitivity of 100% for HTLV-1 and 62% for HTLV-2. In our work, for HTLV-1 detection, high diagnostic sensitivity has been observed, while the achieved sensitivity for HTLV-2 diagnosis remains much lower. The lower sensitivity for HTLV-2 in our assay might be related to several reasons, despite the effort of an additional accessory designed reverse primer (HTLV-2 Rev2*_pol*, see Table 1). The possible reason might be that the HTLV-2 proviral load, in general, is lower compared to HTLV-1 thus leading to lower sensitivity in viral detection (28). Moreover, HTLV-2 can also represent interspecies genetic divergence with the predominance of a specific subtype in our tested population, which can influence the performance of the diagnostic test. The analytic sensitivity for HTLV-2 was higher compared to HTLV-1 (10.9 vs. 3.9 copies/reaction). To address if HTLV-2/HIV-1 infection may affect the test sensibility, positive samples for both viruses were evaluated, and only one of eight samples was missed by the qualitative duoplex qPCR-HTLV-1/2 platform. We believe that neither immune suppression nor antiretroviral treatment for HIV-1 infection was related to this negative result. Similarly, Estes and Sevall noted similar sensibility to HTLV-2 (80%) using a multiplex approach (25). Our qualitative duoplex-RT-PCR-HTLV1/2 assay did not present amplification in all HTLV-1/2 non-infected samples, and thus reached 100% diagnostic specificity. Andrade et al. (29) demonstrated 98.5% of diagnostic specificity indicating the presence of false positivity. In fact, there are several qPCR methodologies for HTLV detection; some of them use SYBR^®^ Green, Molecular Beacons, and TaqMan^®^ probes (30). As mentioned before, these techniques have been developed to detect the most conserved regions of the HTLV 1/2 genome, including genes like *gag, tax* and *pol* (30). The use of different methodologies and target regions could impact directly on the sensitivity of the diagnostic test.

For the multicentric evaluation, we included four of the main clinical HTLV centers in Brazil with primary attendance and follow-up of HTLV-positive individuals. The optimized kit and training program were also provided in each location. The samples tested in this step were previously confirmed as HTLV-positive by another method such as nested-PCR or real-time PCR. As shown in Table 3, we achieved 91.1% of concordance between the tested samples. The discordant cases may be related to differences in the sensitivity of the HTLV PCR diagnosis adopted by each participating center. The highest concordance for HTLV-1 was achieved with Bahia and Para states, 96.5% and 89.3%, respectively, where also real-time PCR was used. In São Paulo city, discordant results were obtained in 20.6% of the cases. Nevertheless in this location the HTLV confirmatory diagnosis is based only on conventional nested-PCR. We also highlight that the majority of discordant cases were related to the presence of HTLV-2, where our assay showed lower detection sensitivity (78.6%) compared to HTLV-1.

Up to now, there is no molecular confirmatory platform for HTLV detection available commercially. The development and the detailed validation of our *duoplex* qPCR technology for confirmatory and discriminatory diagnosis of HTLV-1/2 infection will allow for the confirmation of the positive serological results and can offer a final conclusion to the obtained inconclusive results by EIA or WB. The established multiplex real-time PCR based molecular platform is currently under Brazilian (BR 10.2014.024905.2) and international patent applications (United States of America No. 15/517.449 and European Union patent application No. 158484220).

In conclusion, we developed, optimized and validated a *duoplex* qPCR qualitative test for HTLV-1/2 diagnosis (confirmatory and discriminatory), which is ready-to-use, timesaving and presents low risk for sample cross-contamination. This technique was performed in a unique reaction tube to detect and discriminate between HTLV-1 and HTLV-2. Performance tests showed high sensitivity and specificity compared to the *singleplex* assays and to classical confirmatory methods for HTLV-1/2 diagnosis such as WB. Therefore, this platform can be implemented as a fast HTLV diagnosis and confirmation and to monitor HTLV-infected patients in the clinical centers for the correct management as well as counseling to avoid transmission. Despite the obtained suitable validation parameters of our assay, we highlight that it is important to include more HTLV-2-infected patients and perform the validation processes in other national and international centers engaged in HTLV diagnosis.

## Data Availability Statement

The raw data supporting the conclusions of this article will be made available by the authors, without undue reservation.

## Ethics Statement

The studies involving human participants were reviewed and approved by Clinical Hospital of School Medicine—Process number 20009119.0.1001.5440. The Ethics Committee waived the requirement of written informed consent for participation.

## Author Contributions

MR-J idealized the proposal, performed major experiments and analysis, and wrote the original draft. ER, SS, TA, MP, and DL performed major experiments and analysis and reviewed the original draft. MS supervised sample collection and diagnosis of HTLV-1-infected and healthy individuals. VO performed major experiments and analysis and reviewed the original draft. BG-C, BF, AP, JS, OT, and MS supervised sample collection and diagnosis of HTLV-1-infected and healthy individuals, as well as ambulatory follow-up of HTLV-1 infected individuals. JC, DC, and SK idealized the proposal, supervised and reviewed the original draft. All authors contributed to manuscript revision, read, and approved the submitted version.

## Funding

This work was supported by Financiadora de Estudos e Projetos (FINEP/Process number 1387/10), Centro de Terapia Celular (CTC/FAPESP)/Fundação de Amparo à Pesquisa do Estado de São Paulo (FAPESP—Process number 2013/08135-2), FAPESP n. 2018/07239-2; Conselho Nacional de Desenvolvimento Científico e Tecnológico (CNPq—Processo 465539/2014-9), CNPq n. 301275/2019-0, CNPq n. 301677/2018-2, Fundação Hemocentro de Ribeirão Preto (FUNDHERP); and scholarship from FM/FMUSP to JC.

## Conflict of Interest

The authors declare that the research was conducted in the absence of any commercial or financial relationships that could be construed as a potential conflict of interest.

## Publisher's Note

All claims expressed in this article are solely those of the authors and do not necessarily represent those of their affiliated organizations, or those of the publisher, the editors and the reviewers. Any product that may be evaluated in this article, or claim that may be made by its manufacturer, is not guaranteed or endorsed by the publisher.
